# MOVE UP STUDY RESULTS: WEIGHT LOSS POSITIVELY AFFECTS HEALTH-RELATED QUALITY OF LIFE BUT NOT DEPRESSIVE SYMPTOMS

**DOI:** 10.1093/geroni/igz038.3276

**Published:** 2019-11-08

**Authors:** Elizabeth M Venditti, Robert M Boudreau, Anne B Newman, Michelle E Danielson, Lori A Kieffer, Judith R Rager, Steven Albert

**Affiliations:** 1 Department of Psychiatry, University of Pittsburgh School of Medicine, Pittsburgh, Pennsylvania, United States; 2 The University of Pittsburgh, Pittsburgh, Pennsylvania, United States; 3 University of Pittsburgh Graduate School of Public Health, Pittsburgh, Pennsylvania, United States

## Abstract

Obesity is prevalent among older adults as are increases in depressive symptoms and declines in health-related quality of life (HRQOL). Healthy weight loss and mitigating mild depressive symptoms (MDS) and HRQOL could have critical public health significance. The Mobility and Vitality Lifestyle Program (MOVE UP) led by Community Health Workers delivered 32 healthy aging/weight management group sessions over 13 months. Data from 240 participants were evaluated to assess program impact on CES-D (20-item) depressive symptom and SF-36 HRQOL scores. Participants were 88% female, 28% black/other race, 42% ≥ college-educated. Mean (SD) age was 67.6 (4.1) and BMI was 34.7 (4.7). At baseline, average CES-D score was 7.9 (7.2) and 27.9 % (N = 67) had MDS, scoring 17.1 (6.2). Results show significant mean (SD) weight change of -12.7 (13.3) lb from baseline (p<0.0001). Overall, CES-D mean (SD) score change was -0.4 (6.7) (p=0.33); participants with MDS had an average CES-D decrease of -4.4 (7.8) points (p<0.0001). Further, HRQOL improved significantly in all realms, particularly the physical component score (p<0.0001). SF-36 (SD) total score improved +1.1 (7.6), mental + 2.1 (11.7), and physical + 5.0 (16.7). Regression analyses (age/sex adjusted) demonstrate that for each 5 lb of weight loss there was an average (SEM) 3.35 (1.49) point increase in SF-36 total score (p=0.03). The mitigation of depressive symptoms in the MDS subgroup was not significantly associated with weight loss but may reflect other positive effects of the intervention experience. Conversely, positive HRQOL changes appear to be driven strongly by weight loss.

